# Engineering Disordered Metallic Carbonaceous Materials: A Protocol for the Synthesis via Graphene Edge Hydrolysis

**DOI:** 10.1021/acsanm.6c00047

**Published:** 2026-02-27

**Authors:** Katarzyna Donato, Gavin Kok Wai Koon, Sarah Lee, Alexandra Carvalho, Hui Li Tan, Mariana Costa, Paweł Piotr Michałowski, Zuzana Němečková, Petra Ecorchard, Ricardo K. Donato, Antonio Castro Neto

**Affiliations:** † Centre for Advanced 2D Materials, 37580 National University of Singapore, Singapore 117546, Singapore; ‡ J. Heyrovský Institute of Physical Chemistry, Czech Academy of Sciences, Dolejškova 2155/3, Prague 18223, Czech Republic; § Institute for Functional Intelligent Materials (I-FIM), 37580 National University of Singapore, Singapore 117544, Singapore; ∥ Department of Materials Science and Engineering, 37580 National University of Singapore, Singapore 117575, Singapore; ⊥ Łukasiewicz Research Network, Institute of Microelectronics and Photonics, 02-668 Warsaw, Poland; # Institute of Inorganic Chemistry, Czech Academy of Sciences, Husinec-Řež 1001, Řež 25068, Czech Republic; ∇ Department of Physics, 37580 National University of Singapore, Singapore 117551, Singapore

**Keywords:** 2D materials, graphene oxide, hydrolysis, carbon materials, conductivity, anisotropy

## Abstract

This protocol is a comprehensive account of the intricate processes involved in the rational design, synthesis, and characterization of anisotropic metallic carbon materials. The materials were derived through the hydrolytic oxidation of graphene sheets, followed by self-assembly and mild annealing. The resulting products are highly percolated carbon networks that preserve the essential basal area of the source graphene. Structured into various sections, this document aims to furnish detailed insights crucial for supporting further investigations into these carbon materials. In particular, it highlights the key distinctions from conventional graphite/graphene oxidation protocols, offering a deeper understanding and ensuring the reproducibility of our seminal findings. We believe this differentiation is crucial to preventing the generalization of these materials from the outset, a limitation widely reported in the graphene oxide family and a major source of their inconsistencies, particularly in commercial products.

## Graphical abstract

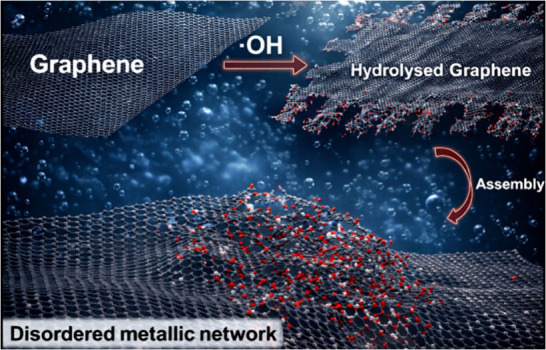

## 1 Introduction

Graphene and graphene oxide (GO), though both derived from graphite, represent structural and functional extremes^1^ and are often incorrectly treated as closely related materials. Graphene is a crystalline, nonpolar, and highly conductive two-dimensional material composed entirely of fully conjugated sp^2^ carbon atoms, and it can be produced through both top-down^2^ and bottom-up^3^ approaches. However, it lacks the processability required by many important technological demands,^4,5^ making the oxidation of graphite^6^ or graphene^7^ into GO an appealing solution.^8^ In contrast, GO is a largely amorphous, polar, and electrically insulating material consisting of a heterogeneous network of sp^2^ and sp^3^ carbon atoms decorated with oxygen-containing functional groups. To partially restore graphene-like properties, particularly transport properties, GO typically requires additional thermal or chemical reduction steps to yield reduced graphene oxide (rGO).^9,10^ However, the oxidation of graphite and graphene is commonly performed under harsh, poorly controlled conditions, leading to stochastic, irreproducible functionalization. Such variability is often exacerbated by the use of reaction additives intended to improve control, which can simultaneously introduce metallic impurities, heteroatom-derived functionalities, carbon vacancies, and radical defects.^10,11^ These factors pose significant challenges for quality control, especially in commercial products,^11^ making it both a rich and chaotic field of investigation.^12^

For this reason, the crucial roles of individual process parameters have only recently been unveiled, such as the different reactivity of graphite and graphene with oxidative species^13,14^ or the influence of water in their oxidative processes.^15,16^ The former highlights how the number of stacked layers affects the nature of the oxidation products, while the latter reveals that the presence of water alters both the reactivity and the functionalization of the final material. Neglecting even these two parameters alone accounts for many of the current inconsistencies in commercial GO products.^11,17,18^ However, understanding and exploiting them can not only enhance reaction control and selectivity, but also enable more refined techniques, such as controlled hydrolysis, long used in advanced oxidation processes for the degradation of aromatic compounds.^19^

GOs derived from the most well-known oxidation methods, i.e., Hummers,^20^ Staudenmaier,^21^ Hofmann,^22^ and Brodie,^23^ present four dominant functional groups (hydroxyl, carboxyl, carbonyl, and epoxy). They are randomly distributed throughout the graphitic structure and vary in composition, especially with the degree of oxidation (add ref review GO). However, using controlled oxidative hydrolysis, we have prepared and explored a different type of graphitic material that shares characteristics with both graphene and GO. It allows for a much more chemically homogeneous oxidation of graphene (almost exclusively hydroxyl functionalization) and selective (most functionalization and defects are toward the edges) (Scheme 1). This is achieved by exploiting an almost-disregarded edge/basal plane reactivity difference under certain conditions. By unveiling these parameters, we selectively modify graphene, increasing its stability in polar solvents, such as water. We also demonstrate the consequences of this increased processability, without losing graphene’s physical properties, such as thermal and electrical conductivities, by forming highly ordered conductive films that do not demand harsh post-treatments.

**Scheme 1:**
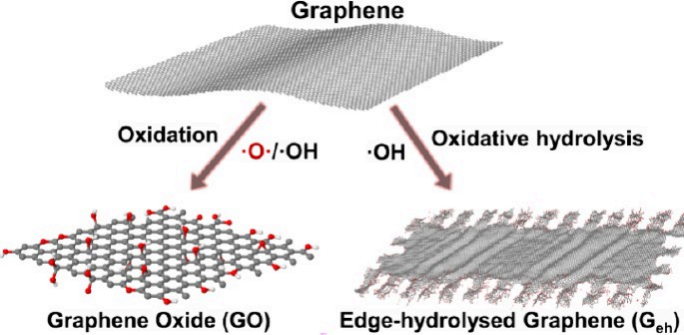
Structural Differences of Oxidized (GO) and Hydrolytically Oxidized Graphene (G_eh_)­^a^

a GO is randomly oxidized and presents four major functional groups (epoxide, carboxyl, carbonyl, and hydroxyl), resulting from the abundant presence of superoxide and hydroxyl radicals. In contrast, G_eh_ almost exclusively presents hydroxyl groups, derived from hydroxyl radicals at the hydrolyzed edges.

In our seminal account on these systems, we have examined structural percolation alongside their mechanical and in-plane thermal and electronic transport properties,^1^ followed by a detailed investigation of anisotropic and temperature-dependent transport and the underlying transport mechanisms.^24,25^ Building upon these foundational studies, we herein present a comprehensive synthesis and characterization protocol, with particular emphasis on scalable and reproducible production. The present discussion is closely integrated with our previous reports, expanding key aspects of their analyses, while distinguishing materials obtained via oxidative hydrolysis from conventional GO. This differentiation clarifies both processing–structure relationships and the origins of the distinct functional properties observed (Scheme 1).

## 2 Experimental Section

### 2.1 Simulations

#### 2.1.1 Density Functional Theory (DFT) Calculations of Graphene Hydrolysis

First-principles calculations were based on the framework of DFT, as implemented in Quantum ESPRESSO,^26^ with the PBE^27^ exchange and correlation functional. Ultrasoft pseudopotentials of the RRKJUS type were used.^28^ We employed a plane wave basis set with kinetic energy cutoffs of 40 Ry for the wave functions. The van der Waals interactions were described using the potential of Grimme.^29,30^ Both supercell and nanoflake models were used. The flakes had 16 carbon atoms per layer, whereas the supercell model contained 32 atoms per layer. For the latter, the Brillouin zone was sampled using a Γ-centered 6 × 6 × 1 Monkhorst–Pack grid.^31^ A supercell periodicity of 30–40 Å in the direction perpendicular to the layers was used to avoid spurious interactions between replicas.

#### 2.1.2 Molecular Dynamics Simulation of Interactions among Edge-Hydrolyzed Species

Molecular dynamics simulations were performed using the LAMMPS code.^32^ The interatomic interactions were modeled using the classical reactive force-field potential of Chenoweth et al.^33^ The graphene flakes were semi-infinite ribbons with serrated edges, composed of 9234 atoms, which were initially created using Jmol.^34^ The interaction between flakes was achieved by imposing periodic boundary conditions, such that the nanoribbon repeats itself along the directions perpendicular to the edges, thus allowing for edge-edge interactions. Additionally, we performed similar calculations for a four-layer graphene flake in vacuum, for comparison between thin sheet clusters and infinitely thick ones.

### 2.2 Preparation of Samples

The substrates (Si, Si/SiO_2_, or Si/Au) used for the characterization of isolated flakes and flake clusters were washed by immersion in acetone (99.8%, Sigma) and isopropyl alcohol (99.8%, Sigma) under sonication (5 min each) and thoroughly dried with N_2_(g). Then highly diluted water dispersions of G_eh_ (<0.01 mg/mL) were drop-casted onto them. These substrate-deposited samples were investigated by a series of microscopy and spectroscopy techniques, as described below.

Bulk compositional characterizations were performed directly on solvent-cast, free-standing films. Film thicknesses ranging from approximately 4 to 400 μm could be obtained. Films thinner than 4 μm were too fragile to be handled, whereas films thicker than 100 μm exhibited increased surface roughness, which adversely affected surface-sensitive measurements. Consequently, all characterizations were carried out using films with an average thickness of approximately 30 μm, which provided mechanical robustness while maintaining flat and homogeneous surfaces.

Prior to solvent casting, the dispersions used for obtaining films were prepared in a 1:1 (v/v) isopropyl alcohol/water solvent mixture with a G_eh_ concentration of approximately 4 mg/mL. This solvent mixture was selected because it enables broad tuning of surface tension and viscosity. Isopropyl alcohol is approximately twice as viscous as water while exhibiting a surface tension nearly 3 times lower. As a result, increasing the water content enhances dispersion stability; however, the higher surface tension promotes droplet formation, which hinders the formation of thin, uniform films. In contrast, the addition of isopropyl alcohol lowers the surface tension while increasing viscosity, suppressing droplet formation without inducing precipitation. With respect to dispersion concentration, even highly concentrated, paste-like dispersions (>15 mg/mL) were capable of forming free-standing films. Nevertheless, dispersions with concentrations above ∼8 mg/mL produced films with progressively increasing surface roughness. Consequently, a concentration of 4 mg/mL was selected as optimal, yielding films with the desired thickness of approximately 30 μm.

The dispersions were initially centrifuged (in a Hettich Rotina 380) at 3000 rpm for 20 min to remove unstable and aggregated species. When required, the resulting supernatant was further subjected to an adapted isopycnic centrifugation protocol to control platelet size homogeneity. Specifically, the supernatant from the first centrifugation step was centrifuged again at 6000 rpm for 20 min, producing a supernatant with a gradient in platelet lateral size, increasing from the top to the bottom of the tube.

### 2.3 Detailed Characterization Methods

For scanning electron microscopy (SEM), samples were drop-casted directly onto Si and Au-coated Si substrates, and analyses were carried out on a FEI Verios 460L field-emission scanning electron microscope operating at 2 kV. SEM–energy-dispersive X-ray spectroscopy (EDX) mappings were performed using a Zeiss Evo 10, operating at 6.3 kV.

High-resolution transmission electron microscopy (HRTEM) was performed using a Talos F200X equipped with an energy-dispersive X-ray spectrometer for elemental analysis. The sample was aqueous dispersed onto a lacey-carbon-coated gold TEM grid and allowed it to dry under ambient conditions. Imaging was carried out at an accelerating voltage of 200 kV. To minimize beam-induced damage, the probe current was limited to approximately 1 nA during acquisition.

For optical microscopy images of isolated G_eh_ flakes, highly diluted samples (<0.01 mg/mL) were drop-casted onto Si/SiO_2_ substrates and dried at room temperature. Then they were further dried at 40 °C under vacuum and imaged using a Leica DM4000 M microscope.

X-ray diffraction (XRD) measurement of the thin film was performed on a diffractometer (Rigaku Miniflex 600) equipped with a Bragg–Brentano θ/2θ goniometer, using Cu Kα radiation (1.5406 Å) in a 2θ range from 3° to 50°, 0.05°/step, and integration time of 2 s/step. All samples were measured using the same mass (300 mg), and the raw XRD data were plotted using absolute count values as a function of 2θ.

X-ray photoelectron spectroscopy (XPS) was performed using Kratos AXIS Supra^+^. Each spectrum was an average of five scans with an emission current of 150 eV and a step size of 1 eV for survey spectra and an emission current of 20 eV and a step size of 0.05 eV for high-resolution spectra. An ion gun was used during each scan to neutralize the charging phenomena. Data analysis and fitting were performed with ESCApe software after Shirley background subtraction. The deconvolution of the C 1s peak was performed considering an asymmetric nature of aromatic sp^2^ components fitted (asymmetry parameter = 0.14). The contributions of all other functional groups and the sp^3^ C 1s signals were fitted using standard symmetric Gaussian and Lorentzian curves.^35^

Secondary-ion mass spectrometry (SIMS) measurements were performed using a CAMECA IMS SC Ultra instrument. A cesium primary beam with a high impact energy (16 keV) and a high current density (450 nA for a 50-μm-diameter beam) was employed to acquire full depth profiles of carbon-based films. The beam was rastered over an area of 250 × 250 μm^2^, while the analysis area was restricted to 100 × 100 μm^2^. The primary ions impinged the surface at an angle of 51°, and to minimize the shadowing effect, the analysis area was shifted by 70 μm along the direction of the incident beam. All signals were recorded in the positive secondary-ion mode as Cs_2_X^+^ cluster ions and normalized to the Cs_2_^+^ signal. This acquisition mode is known to significantly reduce matrix effects and enables semiquantitative analysis of light elements such as carbon and oxygen.^36^ Carbon concentration calibration was performed using a reference highly oriented pyrolytic graphite (HOPG) sample, where the Cs_2_C^+^/Cs_2_^+^ intensity ratio was assigned to 100 atom % carbon. The detection limit for carbon was determined to be 0.0078 atom %. Oxygen calibration was based on a HOPG reference sample implanted with oxygen ions. A representative oxygen implantation profile is shown in Figure S1. The detection limit for oxygen was determined to be 0.0097 atom %, corresponding to a single count of the Cs_2_O^+^ signal. The average Cs_2_^+^ intensity was identical for both reference measurements, allowing a direct comparison of normalized signal ratios. Based on the semiquantitative nature of the Cs_2_X^+^/Cs_2_^+^ detection scheme, a linear scaling between signal ratios and elemental concentrations was assumed. The validity of this assumption was verified by monitoring the sum of the determined carbon and oxygen concentrations, which remained within 100 ± 1% for all depth points and all analyzed samples, despite independent calibration of both elements. The dominant sources of uncertainty are therefore associated not with instrumental sensitivity or counting statistics but with the underlying assumptions of linear signal scaling and non-negligible mutual influence of high oxygen concentration on the measured carbon signal. These effects were minimized by employing Cs_2_X^+^ cluster ions rather than CsX^+^, a well-established approach for reducing matrix effects in SIMS measurements.^37^ Each sample was measured twice, once from each side, following a frontside and backside SIMS configuration. The resulting depth profiles were in full agreement, demonstrating excellent reproducibility and excluding beam-induced damage, charging effects, or depth-dependent measurement artifacts. The strategy for reliable depth profiling of thick samples, including mitigation of shadowing and long-term beam stability, has been described in detail elsewhere^38^ and was applied throughout the present study.

A simultaneous thermogravimetric analysis/differential scanning calorimetry (TGA/DSC) analyzer SDT 650 (TA Instruments) calibrated with sapphire and zinc standards was used to study the thermal behavior of the materials. Film samples (∼15 mg) were placed in a ceramic pan (90 μL) with the punctured lid and heated at a constant rate (10 °C/min) under a synthetic air atmosphere (100 mL/min). An empty ceramic pan was used as a reference. During heating, the instrument simultaneously measured changes in the sample weight and heat flow. The obtained curves were processed using TRIOS software (TA Instruments). Sample triplicates, with standard deviation, are presented in Figure S2.

Atomic force microscopy (AFM) topology images were acquired in a Bruker Dimension Icon microscope operated in tapping mode, with scan lines of 512, and the height profile images were obtained using the open-source AFM image processing tool Gwyddion.

Confocal Raman spectroscopy was carried out in a Witec Alpha 300R, with an excitation wavelength of 532 nm and a 100× objective with a numeric aperture of 0.9. The spectra were normalized with respect to the G-band intensity.

### 2.4 Mechanical Properties of Films

Through-plane nano- and micromechanical properties of films, before and after annealing, were investigated using AFM (Bruker Icon PeakForce), Peak Force Quantitative Nanomechanics (QNM), in air mode. The tips used were RTESPA-300 and RTESPA-525 models, with spring constants *k* = 40 and 200 N/m, respectively. The estimated tip radii were between 8 and 12 nm in both cases, and the maps were acquired with high resolution (512 samples/line and 512 lines per image). After maps were collected, the images from different channels were analyzed using the software NanoScope Analysis. Dynamic mechanical analyses (DMA) were also performed, using a DMA850 (TA Instruments) at a fixed frequency (1 Hz) and strain amplitude (0.01%) using a thin film clamp in the tensile mode. The rectangular films 10.0 × 8.0 × 0.1 mm (*L* × *W* × *T*) were cooled at 3°/min using a gas cooling accessory filled with liquid nitrogen.

### 2.5 Thermal Transport of the Films

Thermal images of G^0^ films were captured using a Tix500 thermal camera (Fluke, Everett, WA, USA) and treated using SmartView Classic 4.4 software with the reference emissivity set to 0.8 (in the range of purified carbon materials), the relative humidity set to 50%, and the environment temperature was set to 21 °C (preset conditions in the laboratory). Thermal images of rectangular strips were measured using a hot plate as the heat source, with the temperature set to ∼150 °C. Due to its low infrared emissivity (∼0.03), a polished Cu film was used to cover the heat source to prevent the transmission of background infrared emission. Specimens measuring 10 × 80 mm were prepared and placed directly onto the heated Cu sheet, and thermal images were captured upon thermal stabilization (∼20 min). The G^0^_(6a)_ film strip (after annealing) was compared to two commercial carbon/graphene-based thermoconductive films, graphene-based (T_G(film)_) and graphite-based (T_Gr(film)_), and a nonconductive paper strip (α ∼ 0.07 mm^2^/s). The setup for the thermal imaging is demonstrated in Figure S3a.

Thermal imaging measurements were also performed on a 5-in. solvent-cast film (Figure S3b), before (G^0^_(6)_) and after (G^0^_(6a)_) annealing, using a heat source with the temperature set to 200 °C. Thermocouples were set 1 in. apart from the heat source to compare the conducted heat with the radiation heat captured by the thermal camera (Figure S3c). The film emissivity at thermodynamic equilibrium was obtained using the Stefan–Boltzmann law, *P* = εσ*T* ^4^, where ε is the film emissivity, σ is the Stefan–Boltzmann constant, and *T* is the surface temperature. The integrated emissivity ε of the surface is ε = ε_I_(*T*_I_/*T*)^4^, where ε_I_ is the emissivity used for thermal imaging (0.8), *T*_I_ is the infrared temperature (25 °C), and *T* is the temperature measured with the thermocouple (56 °C), resulting in ε = 0.03 for G^0^_(6a)_. ε was also measured for G^0^_(6)_, ε ∼ 0.1; however, this value is not trustworthy because annealing takes place during the measurement and both radiation and conduction heat-related temperatures are unstable.

Laser flash analysis (LFA) was used to characterize the anisotropic thermal diffusivity of the films, as it is commonly used to measure highly thermally conductive thin films. Free-standing samples with thickness in the range 50–400 μm were precut into a circular shape with a diameter of ∼25.4 mm before loading into the standard through-plane and customized in-plane sample holders for the Netzsch LFA 467 system. The thickness of each sample was measured at multiple radial positions across the disk using a calibrated micrometer, and the mean value was used for analysis. The standard deviation of thickness was included in the uncertainty analysis. The measurements were performed with the Netzsch LFA 467 system using a standard model, which is a modified version of the Cape and Lehman model considering both radial and axial heat losses.

#### 2.5.1 Contact Resistance Correction

LFA is an optical, noncontact transient technique. Heat is introduced via a laser pulse, and the temperature is detected radiatively by an IR detector. The specimen is not part of a steady-state heat-flow stack, and therefore no thermal contact resistance correction is required, unlike steady-state guarded hot plate or TIM measurements. Measurements were repeated at multiple shots to confirm reproducibility.

#### 2.5.2 Out-of-Plane (Through-Thickness) Diffusivity, α_⊥_

Measured in the standard Netzsch front-face laser/rear-face IR detection configuration. A short laser pulse heats the front surface and the transient temperature rise of the rear surface is recorded by an IR detector (Figure S3d). Diffusivity was extracted using the Netzsch Proteus software, applying the Cowan model with heat-loss correction and finite pulse correction.

#### 2.5.3 In-Plane Diffusivity, α_∥_

In-plane thermal diffusivity was measured using the Netzsch lateral/radial heat-flow method, in which the laser pulse excites a defined region of the disk and the temperature response is detected at a lateral offset (Figure S3d). The transient response is fitted using the Netzsch in-plane heat diffusion model implemented in Proteus. This model accounts for radial heat spreading, finite pulse duration, and heat losses.

In addition, the in-plane model for calculation of the diffusivities takes into consideration our sample’s anisotropic nature, whereby the back surface of the sample is heated by a xenon (higher power) with a predefined pulse width (Figure S3d). The temperature rise signal *T*_(t)_ is monitored on the top surface with the help of an IR detector (typically InSb or MCT). The thermal diffusivity of the sample is given by

$$\lambda =0.1388\frac{d^{2}}{t_{1/2}}$$

whereby *d* is the thickness of the thin film sample and *t*_1/2_ is the half the time it takes for the temperature to rise to the maximum temperature. This simplified model assumes an isotropic and adiabatic system.

The specific heat capacity (*C*_p_) of our films was also measured using the laser flash comparison method, by contrasting it with a reference material that possesses a known specific heat capacity.^39^ To achieve accurate results, the reference sample must have a comparable cross-sectional shape and thermal conductivity values and be suitable for the temperature range being studied (e.g., graphite). Here, we measured the value of *C*_p_ for our films starting from room temperature up to 300 °C. As plotted in Figure S3e, the value increases almost linearly from 1.8 to 2.3, with increasing temperature. These *C*_p_ values from Figure S3e were used to calculate the thermal conductivity presented in Figure S3d.

### 2.6 Electronic Transport of the Films

Sheet resistance (*R*_s_) is a common electrical property used for the characterization of conducting and semiconducting uniform thin films. The main advantage of this parameter is that it is independent of the sample size, and it can be measured directly via a 4-point probe method.

$$R_{s}=\frac{\rho }{t}$$

It is defined as the resistivity (ρ) of a material divided by its thickness (*t*), with ohm (Ω/sq) units.

The most common method for measuring in-plane sheet resistance is by employing the 4-point probes setup to eliminate the contact resistance (Figure S4a). A direct current is applied between the outer two probes and a voltage drop is measured between the inner two probes. The sheet resistance can then be calculated using the following equation:

$$R_{s}=\frac{\pi }{\ln\left(2\right)}\frac{\Delta V}{I}$$

This equation is valid only if the thickness of the material being tested is less than 40% of the spacing between the probes (*d* = 1 mm) and the lateral size of the sample is sufficiently larger. Otherwise, other geometric correction factors are required to account for the size, shape and thickness of the sample, which are included in the dasoleng measurement setup. Thus, the same batch of samples used for thermal diffusivity measurements, without further preparation, was used for sheet resistance measurements using a dasoleng 4-probe measurement setup.

For the through-plane measurements, a 4-point probe device is also used to measure the electrical conductivity of the film to eliminate contact resistance. However, a device was fabricated on a 4-in. SiO_2_ (285 Å)/Si wafer. First, the bottom layer of metal contacts, which consists of Ti (50 Å)/Au (1000 Å), was deposited via an electron-beam evaporator system (AJA ATC-E) with the aid of a thermal tape mask. Next, G_eh_ was drop-casted onto a predefined square with an area of 20 mm × 20 mm. The sample was left to dry in a fume hood for few hours. Finally, the top layer of metal contacts, which consists of Au (1000 Å), was deposited using the same method as described above. Thus, the device’s metal electrodes deposited on the bottom and top sandwich the film (Figure S4b,c).

A sinusoidal alternating current is applied between a pair of bottom and top contacts, and the resultant voltage drop is measured between the remaining pair with a lock-in amplifier (Stanford Research SR830). The resistivity (conductivity) can then be calculated using the following equation:

$$\rho =\frac{\Delta V}{I}\frac{A}{t}$$

## 3 Results and Discussion

### 3.1 Simulations

DFT and molecular dynamics simulations were used to better understand the edge-hydrolysis reactions and the subsequent interactions and self-assembly among the produced edge-hydrolyzed species, respectively. The methods and parameters used are described in detail in the Detailed Characterization Methods section.

Using first-principles calculations based on the framework of DFT, we have determined the reaction enthalpies (Δ*H*_r_) of hydroxyl groups with different functional groups at the edges of graphene, at its basal plane, or at defect sites, which corresponds to the difference between the total energies of reagents and products (Tables 1 and 2). A negative value indicates energy release. To respect charge balance, some reactions require the transfer of an electron either to the graphene or to a defect. Thus, we have modeled some of the reactions in the presence of either an epoxy or a hydroxyl radical, both of which can become negatively charged. When negatively charged, the epoxy relaxes to a carboxyl configuration.

**Table 1:**
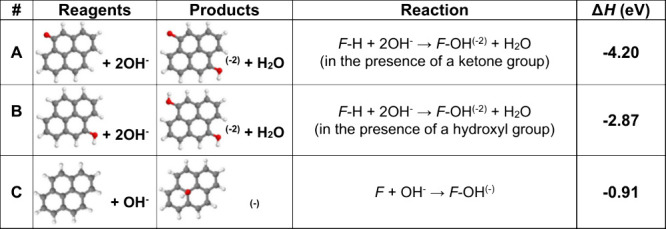
Reactions with Finite Graphene Flakes (*F*) and Respective Reaction Enthalpies, Calculated Using DFT

**Table 2:**
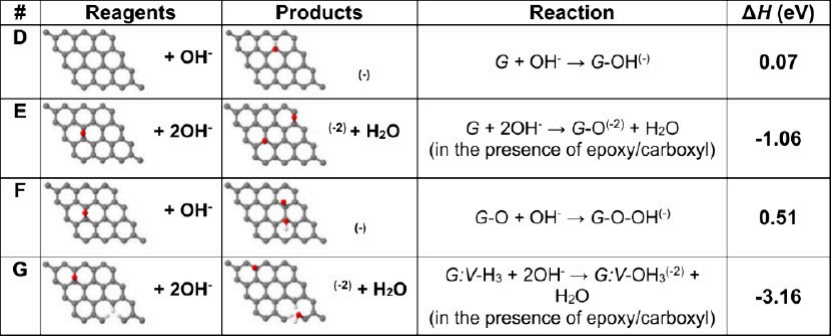
Reactions with Infinite Graphene Sheets (G), with or without Defects, and Respective Reaction Enthalpies, Calculated Using DFT

As we discuss elsewhere,^24^ there are three different regions with distinct reactivities that are dependent on the thickness and lateral size of the graphitic precursor, and the Δ*H*_r_ differs considerably at the edge and basal planes of the exterior layer. The edges present a considerably negative Δ*H* and occur spontaneously independent of the temperature, unlike the reactions at the basal plane that are more sensitive to temperature, and this selectivity increases proportionally with increasing lateral size (see Figure 1d in ref 24). Although it is known that the integrity of the graphene used as a precursor is important, because this selectivity may be affected by defects in the basal plane,^40,41^ we observed that basal defects and functional groups are only minor contributors affecting the selectivity (Tables 1 and 2). Altogether, edge functionalization with ^•^OH is prioritized in all cases studied, including finite and infinite structures, with or without defects. As a consequence, at lower temperatures (*T* < 10 °C), selectivity could be achieved even with defective graphene.

Molecular dynamics simulations were used to give us a better understanding of the interactions among edge-hydrolyzed species, the self-assembly process, and the final structure of the self-assembled films. For that, periodic boundary conditions were applied along the direction perpendicular to the edges, allowing parallel edges of neighboring nanoribbon images to interact in-plane (Figure 1). Additionally, we have also constructed a 3D model where the repetition along the direction perpendicular to the basal plane allowed the edges to interact with both the adjacent graphene ribbon images above and below (Figure 1c).

**Figure 1:**
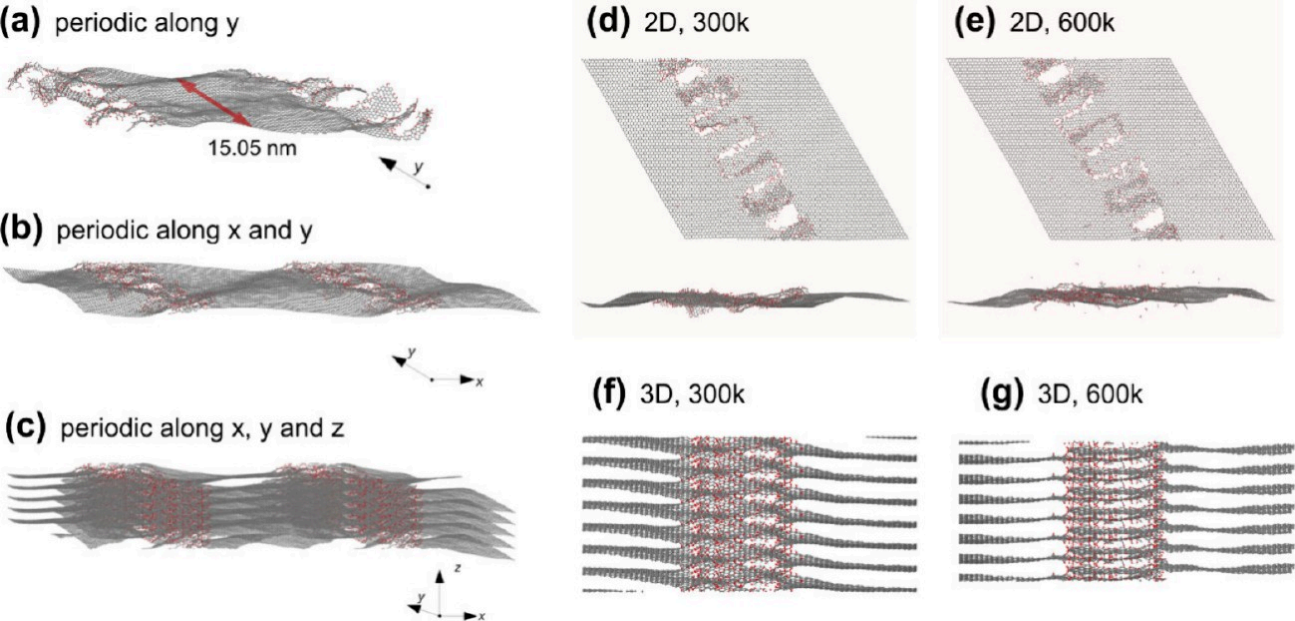
Scheme of the periodic model used to simulate in-plane and out-of-plane edge interactions between G_eh_ layers: (a) isolated flake; (b) 2D model; (c) 3D model. Molecular dynamics simulations of interacting G_eh_ species as a function of the temperature: (d–e) 2D model at 300 and 600 K, top and side views; (f–g) 3D model at 300 and 600 K, side view (a slice is shown for clarity).

Molecular dynamics simulations were used to give us a better understanding of the interactions among edge-hydrolyzed species, self-assembly process and the final structure of the self-assembled films. For that, periodic boundary conditions were applied along the direction perpendicular to the edges, allowing parallel edges of neighboring nanoribbon images to interact in-plane (Figure 1). Additionally, we have also constructed a 3D model where the repetition along the direction perpendicular to the basal plane allowed the edges to interact both with the adjacent graphene ribbon images above and below (Figure 1c). After initial optimization and thermalization, the annealing of the flakes was simulated within the isothermal–isobaric ensemble for 0.4 ns at 300, 400, 500, and 600 K, at a pressure of 0 atm, using a Nosé–Hoover thermostat style integration, with an integration time step of 0.1 fs. The *y*-direction cell dimension was kept fixed during the calculations, while the periodic *x* (or *x* and *z*) direction were allowed to relax in the 2D (3D) models. The cell dimension along the *x* direction was optimized to minimize the in-plane pressure at 300, 400, 500, and 600 K, yielding realistic edge reconstruction, with the degree of covalent bonding increasing with temperature (Figure 1d,e). Periodic repetition along the *z* direction under the same temperature conditions yields a 3D system where the flake edges can interact and bond with the flakes above and below, forming a highly anisotropic structure (Figure 1f,g).

Oxygen and hydroxyl groups were placed predominantly in the neighborhood of the edges, with an O/H ratio of ∼1.5, similar to the values obtained experimentally (Table 6). The simulations show that at 300 K the edges of neighboring graphene flakes align (i.e., the edges of neighboring nanoribbon periodic images, in the simulations). However, above 500 K, significant cross-linking takes place, both “zipping” adjacent edges in the same plane together and allowing edge fragments to connect to the flakes above and below, forming a 3D structure. These parameters serve as guides for the experimental processes applied in Section 3.2.

### 3.2 Edge Hydrolysis and Film Formation

#### 3.2.1 Graphene Sources

To prospect the generality of the process, three commercial sources of graphene nanoplatelets (GNPs) were functionalized using the G_eh_ platform. As we do not believe it is relevant for the process, we do not reveal their names, but their structural properties, carbon material quality-related properties, and elemental analysis are summarized in Tables 3 and 4. We must highlight that the three graphene sources used below were tested, and two of them yielded good quality products (GNP 1 and GNP 3), while GNP 2 led to less reliable and lower quality products (dramatic decrease in selectivity). The three precursors vastly differ in many characteristics, including the level of defectiveness and oxygen content; however, we observed that the most relevant parameter to achieve edge selectivity is the number of layers (DL50 and DL90). Although the process is very tolerant to relatively thick flake stacks, there seems to be a thickness threshold where selectivity is lost, probably related to dispersion stability during the reaction. For this reason, further investigations and the results presenting different levels of oxidation were focused on GNP 3.

**Table 3:** Relevant Carbon-Related Characterizations of Graphene Sources Used for G_eh_ Preparation^a^

	flake size (μm)		no. of layers		flake quality		carbon composition (%)				
GNP	DF_50_	DF_90_	DL_50_	DL_90_	*I*_G_/*I*_2D_	*I*_D_/*I*_G_	C sp	C sp^2^	C sp^3^	C–O	C=O
GNP 1	>0.5	>1.1	<10	<50	∼1.9	∼0.4	3.32	55.43	16.03	18.88	6.34
GNP 2	>0.5	>1.1	<30	<300	∼2.50	∼0.3	0	66.10	20.02	6.07	7.80
GNP 3	>0.8	>1.8	<10	<30	∼2.83	∼0.2	0	78.69	12.16	6.00	6.34

a DF_50_ and DL_50_ are the median values for the flake lateral size obtained via optical microscopy and the number of layers obtained via AFM, respectively. DF_90_ and DL_90_ are obtained in the same manner, but their values indicate where 90% of the distribution lies. *I*_G_/*I*_2D_ and *I*_D_/*I*_G_ are obtained via Raman spectrometry. The carbon composition and oxidation are obtained by XPS.

**Table 4:** Elemental Analysis of Graphene Sources Used for G_eh_ Preparation

	elemental analysis (%)					
GNP	C	H	N	S	O	total
GNP 1	78.1	2.1	0	0.1	16.6	96.9
GNP 2	95.7	0.3	0.1	0.1	1.0	97.2
GNP 3	96.6	0.2	0	0.2	3.0	100

#### 3.2.2 Graphene Edge-Hydrolysis Protocol

Our platform allows for highly concentrated, selective edge hydrolysis reactions (up to 5.7%) and, consequently, very high product outputs. The reactions herein presented were performed mainly in two reactor sizes, i.e., a Mettler Toledo Optimax 1001 with 1 L capacity and a scale-up size in-house constructed 20 L thermostat reactor. Using a 1 L reactor as a reference, batches with up to 57 g can be processed in cycles as short as 2 h, resulting in reaction rates up to ∼28 g/Lh (before dilution, details below). The small amounts of sulfuric acid and potassium permanganate used at low temperatures allow gradual hydrolysis from the edges to the center of the graphene sheets, using the previously mentioned differences in enthalpic and entropic effects on the edge/basal plane reactivity.

The basic initiation of the reaction based on sulfuric acid and potassium permanganate is the same as that in the classic Hummers method (eqs 1–3).

1 $${\mathrm{KMnO}}_{4}+3H_{2}{\mathrm{SO}}_{4}\to K^{+}+{{\mathrm{MnO}}_{3}}^{+}+{3{\mathrm{HSO}}_{4}}^{-}+H^{+}+H_{2}O$$

2 $${\mathrm{KMnO}}_{4}\to K^{+}+{{\mathrm{MnO}}_{4}}^{-}$$

3 $${{\mathrm{MnO}}_{3}}^{+}+{{\mathrm{MnO}}_{4}}^{-}\to {\mathrm{Mn}}_{2}O_{7}\left[\mathrm{Mn}\left(\mathrm{VII}\right)\right]$$

However, the presence of water and Mn­(VII) (3:1 molar ratio) leads to the formation of O_3_, which degrades into ^•^O^•^ and especially HO^•^ (by the further reaction of O_3_ with H_2_O) (eqs 4–7).^42^

4 $$3H_{2}O+2\mathrm{Mn}\left(\mathrm{VII}\right)\to O_{3}+2\mathrm{Mn}\left(\mathrm{IV}\right)$$

5 O3→Mn(IV)O2+O••

6 $$H_{2}O+O_{3}\overset{\mathrm{Mn}\left(\mathrm{IV}\right)}{\to }O_{2}+2{\mathrm{HO}}^{•}$$

7 graphene→O3+O••+HO•Geh

These radicals are highly reactive and, when in the presence of graphene, will react promptly, forming a hydroxyl-group-rich structure (see XPS in Figure 3a). For this reason, temperature control is essential to avoid random reactions and to promote selective functionalization. In summary, many functionalization variations can be applied to graphene using simple modifications of the classic chemical oxidation processes, enabling scaling-up the production of interesting new applications of modified graphene such as 2D electrolytes.^43–47^

Thus, concentrated sulfuric acid (H_2_SO_4_, 98% Sigma-Aldrich) is initially added to a reactor and cooled down below 5 °C under constant stirring (∼50 RPM). Graphene is then added to the H_2_SO_4_, where the graphene-to-acid ratio defines the final level of oxidation/functionalization (Figure 2, step 1). The maximum concentration supported by this process (due to viscosity restrictions), which yields the mildest oxidation, is 210 mg of graphene per 1 mL of H_2_SO_4_. After stirring until complete graphene dispersion, forming a black viscous liquid (about 5–10 min), a precooled to 5 °C 5% KMnO_4_ aqueous solution (prepared from 99% ACS reagent, ≥99.0%, Sigma-Aldrich KMnO_4_(s) and water deionized in a Milli-Q system with a resistivity of 18.2 MΩ·cm at 25 °C) is slowly added using a peristaltic pump. The pump flow rate depends on the reactor’s size and cooling efficiency, and was set as the maximum rate while maintaining the temperature constant at around 5 °C, e.g., ∼3–10 mL/min and a stirring speed at 150 RPM for the 1L reactor (Figure 2, step 2). This process takes up to 1 h, depending on the reaction volume and flow applied, with the total amount of KMnO_4_ set to 133 mg per 1 mL of H_2_SO_4_. At this stage, the formation of ^•^OH and ^•^O^•^ radicals occurs; however, their reactivity is reduced due to the low temperature. After KMnO_4_ addition, the temperature is increased to 22 °C for 20 min (hydrolytic oxidation stage) (Figure 2, step 3). Because the level of oxidation can be adjusted for fine-tuning of the properties, Table 5 summarizes the reactant ratios used to obtain the three different C/O ratios described in Table 6 (G_eh(6)_, G_eh(10)_, and G_eh(15)_). For brevity, the samples with varying oxidation are designated according to their C/O ratio, e.g., a G_eh_ with C/O = 6 is referred to as G_eh(6)_ before annealing and G_eh(6a)_ after annealing.

**Figure 2:**
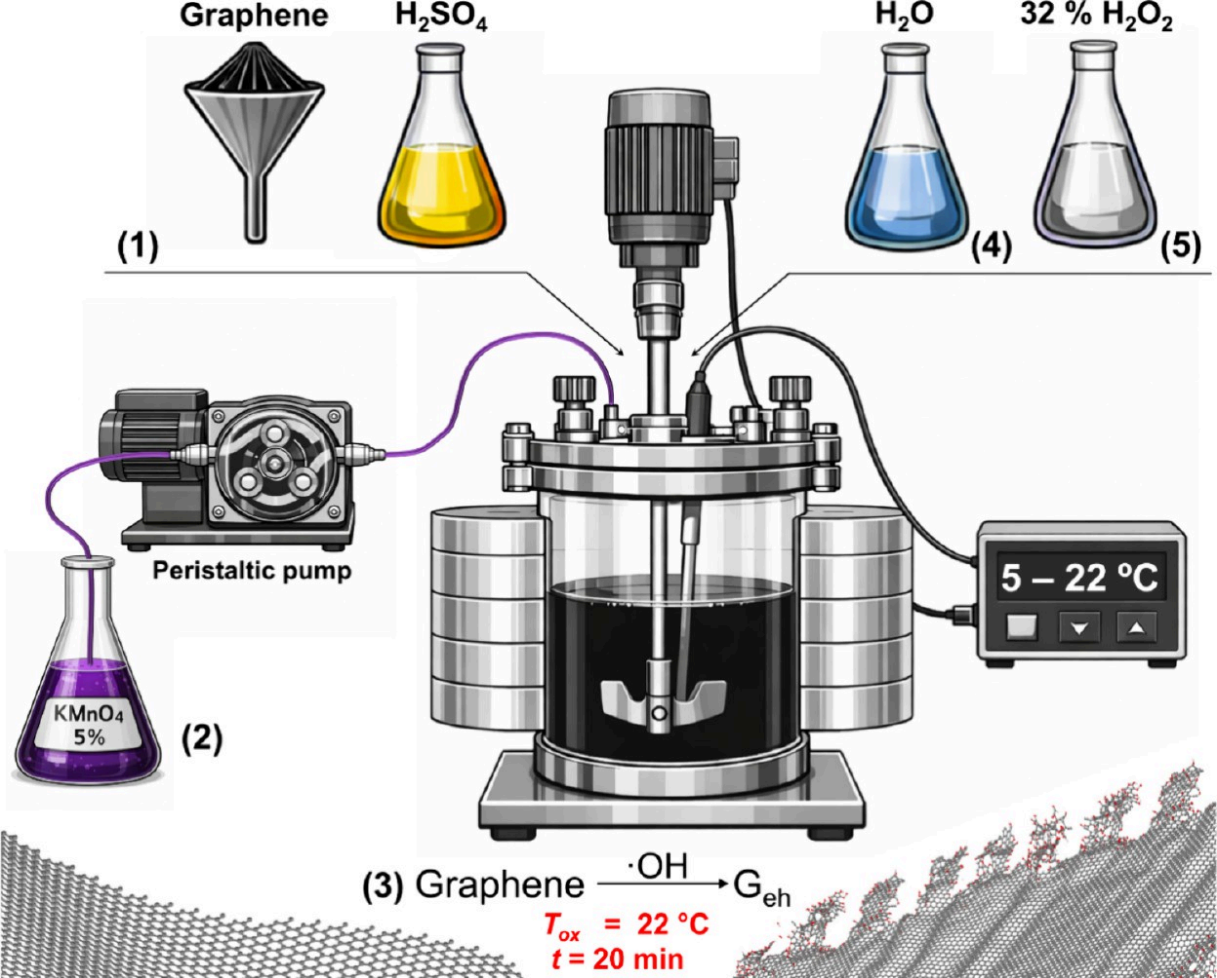
Schematic representation of the selective edge-hydrolysis reaction setup (G_eh_), including the reaction steps: (1) graphene and sulfuric acid addition; (2) potassium permanganate solution addition at low temperature (∼5 °C); (3) temperature increase to 22 °C for ∼20 min (oxidation/functionalization step); (4) dilution with water; (5) reaction quenching.

**Table 5:** Reactant Ratios for Obtaining the Systems with Different Levels of Oxidation

no.	graphene (g)	H_2_SO_4_ (mL)	KMnO_4_ (g)	H_2_O (mL)
G_eh(6)_	1.00	13.60	1.80	34.20
G_eh(10)_	1.00	6.80	0.90	17.10
G_eh(15)_	1.00	4.80	0.64	12.16

**Table 6:**
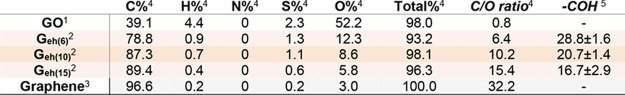
Elemental and Functional Composition of G_eh_ with Different C/O Ratios (G_eh(x)_), also Showing Values for Graphene and GO for Differentiation^a^

a 1: characterized from a commercial GO. 2: value added to *x* in G_eh(*x*)_ refers to the C/O ratio. 3: GNP 3 was used as reference (details in Section 3.2.1). 4: Obtained by elemental analysis. 5: Obtained by XPS (Figure 3a).

Because the temperature is kept low during the radical formation period, the reduced intercalation associated with the overall decreased radical reactivity (but with increased reactivity at the graphene edges relative to the basal plane) greatly favors functionalization at the graphene’s edges. After the reaction, the resulting suspension is cooled to 5 °C, diluted (2 mL of H_2_O per 1 mL of H_2_SO_4_) (Figure 2, step 4), quenched with a 30% H_2_O_2_ solution (Sigma-Aldrich, 0.06 mLH_2_O_2_ per 1 mL 5% KMnO_4_) (Figure 2, step 5), and stirred for 2 h at RT. Then, the resulting suspension is transferred to a separation funnel and left overnight to allow precipitation. For applications requiring higher purity, the precipitated slurry is separated and washed in one cycle using 10% HCl (prepared from 37% ACS-grade Sigma-Aldrich, 7 mL of HCl per 1 mL H_2_SO_4_), and finally dialyzed (SnakeSkin dialysis tubing, Thermo Scientific, 10 kDa molecular molecular-weight-cutoff cellulose membrane) until a stable pH is reached (pH ∼ 6).

#### 3.2.3 Graphene Edge-Hydrolysis Characterization

The proposed reaction mechanism is demonstrated by preparing G_eh_, following the protocol in Section 3.2.2. Product characterizations are presented in Figures 3 and 4. We need to highlight that G_eh_ has a very strong tendency to self-assemble and form organized aggregated structures and films. Thus, to characterize isolated flakes, highly diluted dispersions (<0.01 mg/mL) were prepared and cast onto Si/SiO_2_ substrates to avoid self-assembly during solvent evaporation, and regions with low aggregation were selected. On the other hand, for film characterization, a wide range of dispersion concentrations can be used and high-quality films are formed within a concentration range of 0.01 < *C* < 20 mg/mL.

**Figure 3:**
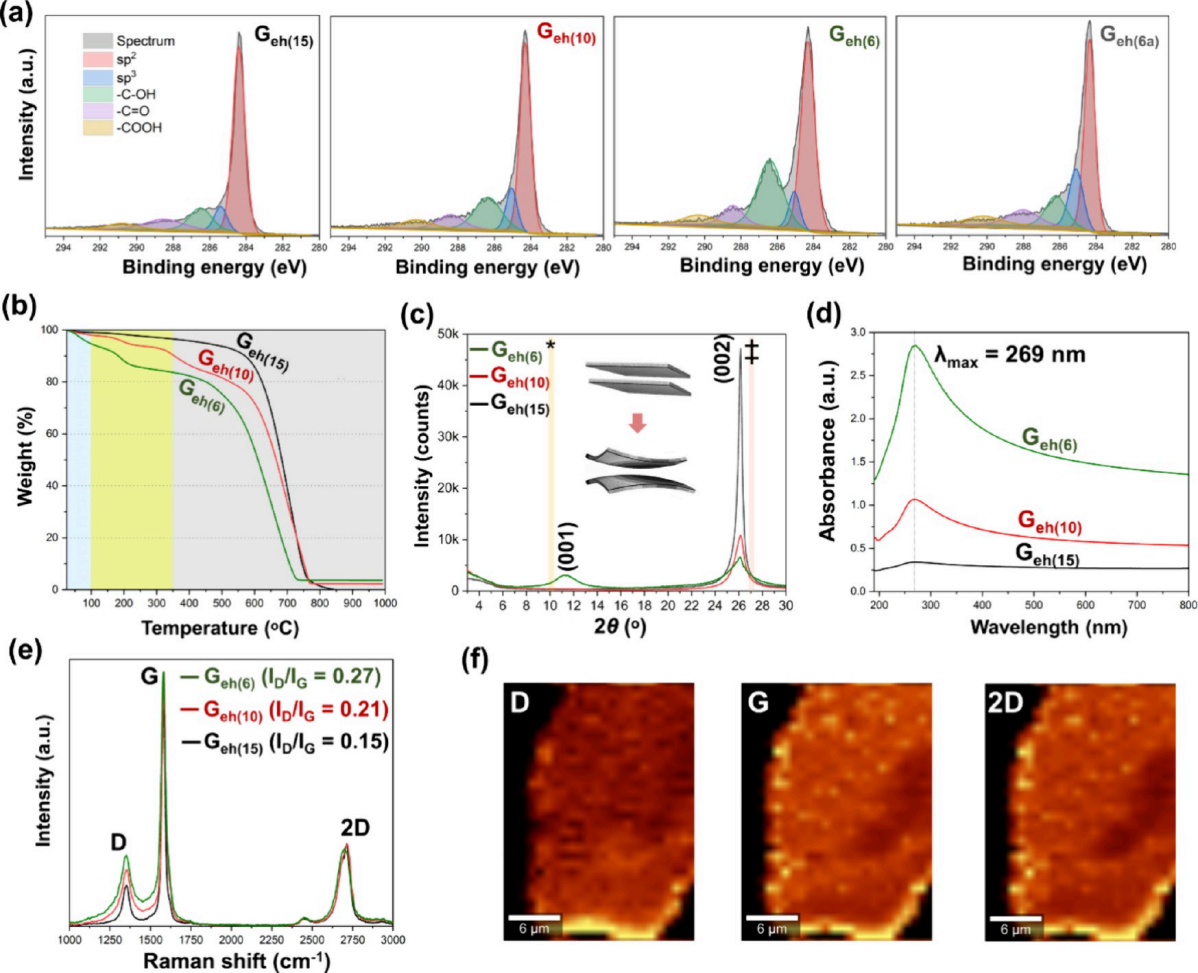
(a) High-resolution XPS demonstrating a dominant C 1s peak and a majority of C–O segments among the oxidation species, which increase with oxidation level (G_eh(15–6)_). The most oxidized sample (G_eh(6)_) was also submitted to annealing at 150 °C (G_eh(6a)_), showing −COH values decreasing to half. (b) TGA highlighting the increase in mass loss at low temperatures with increasing oxidation. (c) Unprocessed XRD curves (with absolute intensity) of G_eh_ with different oxidations, highlighting the expected 2θ angles for GO interlayer spacing (*) and the 002 plane of graphite (‡), demonstrating the absent/shifted peaks for all samples and increased sheet curvature with oxidation. (d) UV–vis of water dispersions of G_eh(6–15)_ with a dominant band at 269 nm. (e) Averaged Raman spectra of G_eh(15–6)_ showing relatively mild changes with varying the oxidation. (f) Raman mappings of an isolated large G_eh(6)_ flake showing the distributions of the intensity of the D, G, and 2D bands.

**Figure 4:**
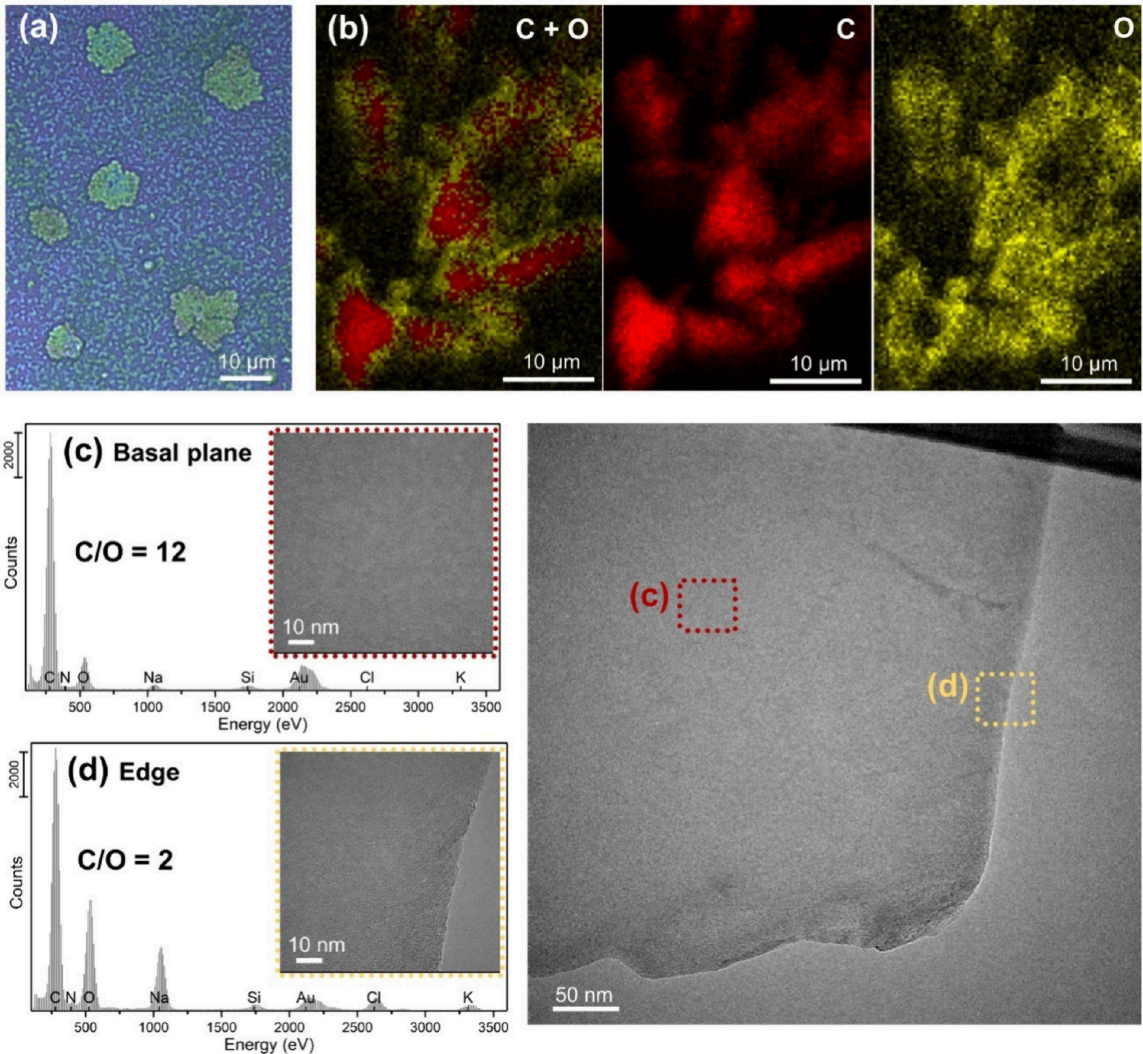
(a) Optical microscopy image (50× magnification). (b) SEM/EDX elemental maps of carbon (C) and oxygen (O). HRTEM/EDX localized elemental analysis of G_eh(6)_ isolated flakes, highlighting the large discrepancy between the (c) C/O ratios at the basal plane (C/O = 12) and (d) edge (C/O = 2).

Very high carbon contents (up to 89% C), mainly represented by C sp^2^ are observed for all samples, even with increasing oxidation (Figure 3a). When the most oxidized sample (G_eh(6)_) is annealed at 150 °C, the number of functional groups decreases to less than half, accompanied by a mild increase in C sp^3^ (Figure 3a), which is associated with the condensation reactions among sheets (see Section 3.2.5). The graphitic basal plane preservation also results in high thermal stability (Figure 3b), with temperatures of maximum degradation (*T*_max_) above 600 °C (under oxidative atmosphere) for all oxidation levels, which are higher than those expected for GO.^48^ The lack of functional groups in the basal plane leads to either the absence (for G_eh(10)_ and G_eh(15)_) or presence of a broad XRD (001) diffraction peak (for G_eh(6)_), which is also shifted to a higher 2θ (i.e., smaller interlayer distance). The (002) diffraction peak is also slightly shifted to lower 2θ, with decreased intensity and broader profile as oxidation increases. This indicates that the restacked layers increasingly curve as the oxidized edge area enlarges (Figure 3c),^49^ which is also more prone to defect-dependent corrugation.^50^ All systems form stable dispersions in water and present dominant π–π* transitions of C sp^2^, observed by the UV–vis absorbance band at λ_max_ = 269 nm (Figure 3d). This absorption, comparable to graphene (∼270 nm) and red-shifted relative to GO (∼230 nm), indicates the presence of extended sp^2^ domains and confirms the preservation of the basal plane.

The Raman profiles are similar among the different systems with varying levels of functionalization (Figure 3e). However, different regions within individual sheets of the same sample show clear variations in Raman profiles, with an increased defect density in areas contiguous with the edges. The Raman maps in Figure 3f illustrate this phenomenon in more detail, by comparing the spatial distribution of intensities of graphene’s fingerprint bands related to the primary mode of the planar sp^2^-bonded carbon (G band), the defect band associated with a ring-breathing mode of sp^2^ carbon rings (D band) and the second order of the D band (2D band). By comparing the color-intensity distributions in the D, G, and 2D maps, the reduced defect density at the basal plane can be clearly evidenced.

Raman profiles also provide us with a defect distribution within the samples, revealing the defined hydrolysis induced by the water-enhanced oxidation (see Section 3.2.2). These hydrolytic defects are also observed in the optical microscopy images of isolated flakes, appearing as fractal-like damage located almost exclusively in the peripheral areas of the flakes (Figure 4a). Moreover, SEM/EDX elemental mapping of C and O throughout the sample clearly shows that these edge defects are associated with an oxidative hydrolysis process (Figure 4b). The elemental maps obtained for G_eh_ show a C distribution coinciding with the G_eh_ aggregates, while the O maps display a pronounced O enrichment at the edges and preservation of the center of the flakes as a result of the mild and selective oxidation of this process. These results are corroborated by the basal plane (Figure 4c) and edge-localized (Figure 4d) elemental distributions obtained by HRTEM/EDX, as well as by the Raman maps for this system (Figure 3f).

#### 3.2.4 Preparation of Self-Assembled Films

After functionalization, the G_eh_ were self-assembled into percolated structures, herein referred to as G^0^. The G^0^ films were prepared by redispersing G_eh_ in a water/isopropyl alcohol mixture (1:1 volume ratio), followed by sonication in an ultrasonic bath (Bandelin Sonorex, 280 W, 35 kHz) for 30 min. The temperature was maintained at 10 °C during sonication to prevent solvent evaporation, using a stainless steel helical coil connected to a recirculating chiller filled with a 1:1 ethylene glycol/water mixture. It is important to highlight that self-assembled films were obtained even without any purification. However, the resulting dispersions were also subjected to an adapted isopycnic centrifugation method,^51^ which allowed us to obtain samples with controllable levels of flake size homogeneity (see details in the Experimental sSection). This was particularly useful for the *Hall-device and the low-temperature electronic transport measurements*, which we discuss elsewhere.^25^

The resulting supernatant is a very homogeneous, shiny black dispersion presenting a liquid-crystal-like appearance. At this stage, the organization of the dispersion seems to be strongly assisted by the interaction between the functionalized regions of G_eh_ and the solvents applied. Although G_eh_ is also stable in pure water, we have noticed that water/isopropyl alcohol mixtures produce faster evaporation and more ordered, stable dispersions. Then, the supernatant was submitted for film formation via direct solvent casting at room temperature onto a Teflon or polypropylene mold, onto copper foils, or alternatively, via vacuum filtration using alumina filter disks (Anodisc, Whatman, 0.02–0.1 μm pore sizes, depending on the lateral size of the source graphene used). Even at room temperature, the solvent evaporation is fast, especially in isopropyl alcohol-containing mixtures, where a large A4-size film forms overnight (∼12 h) at room temperature, or in as little as 2 h when cast onto a copper surface heated to 40 °C. A smaller film can be cast in under 10 min at 60 °C (Video S1).

The highly ordered films can be formed using dispersions with a broad concentration range, from as low as 0.01 mg/mL to as high as 20 mg/mL. However, the optimal concentration for the film formation, defined as the highest concentration yielding impeccable structural order and surface smoothness, is <5 mg/mL. This also reinforces the idea that the high structural order of the final films arises from the material’s anisotropy and interactions (both among sheets and with the solvent), suggesting lower initial entropy and, consequently, a lower energetic cost for the formation of highly ordered films.

#### 3.2.5 Preparation of Films on Complex Surfaces and Confined Spaces

Formation of conductive films on nonflat surfaces, surfaces bearing specific textures, in confined areas is highly demanded in areas addressing the challenges of miniaturization, such as electronics,^12^ where heat management in confined areas is a major issue.^52^ Thus, 250 μL of a 4 mg/mL G_eh(6)_ dispersion in water/isopropyl alcohol (1:1 ratio) was cast onto a SiO_2_-coated Si surface with a 50 × 50 micropillars array (100 μm diameter and 100 μm height pillars over a 100 mm^2^ area), forming a stable droplet. The droplet was left to evaporate (about 30 min) within a fume hood under constant airflow, producing a high-quality 1 mg G^0^ film with a smooth surface (∼4 μm thickness, Figure 5a). To clearly show the thinness and mechanical robustness of the film, the substrate was fractured in half and imaged on top of the fractured surface (Figure 5b). The dotted line in Figure 5a indicates a region between two pillars, corresponding to the area shown in the SEM image in Figure 5b.

**Figure 5:**
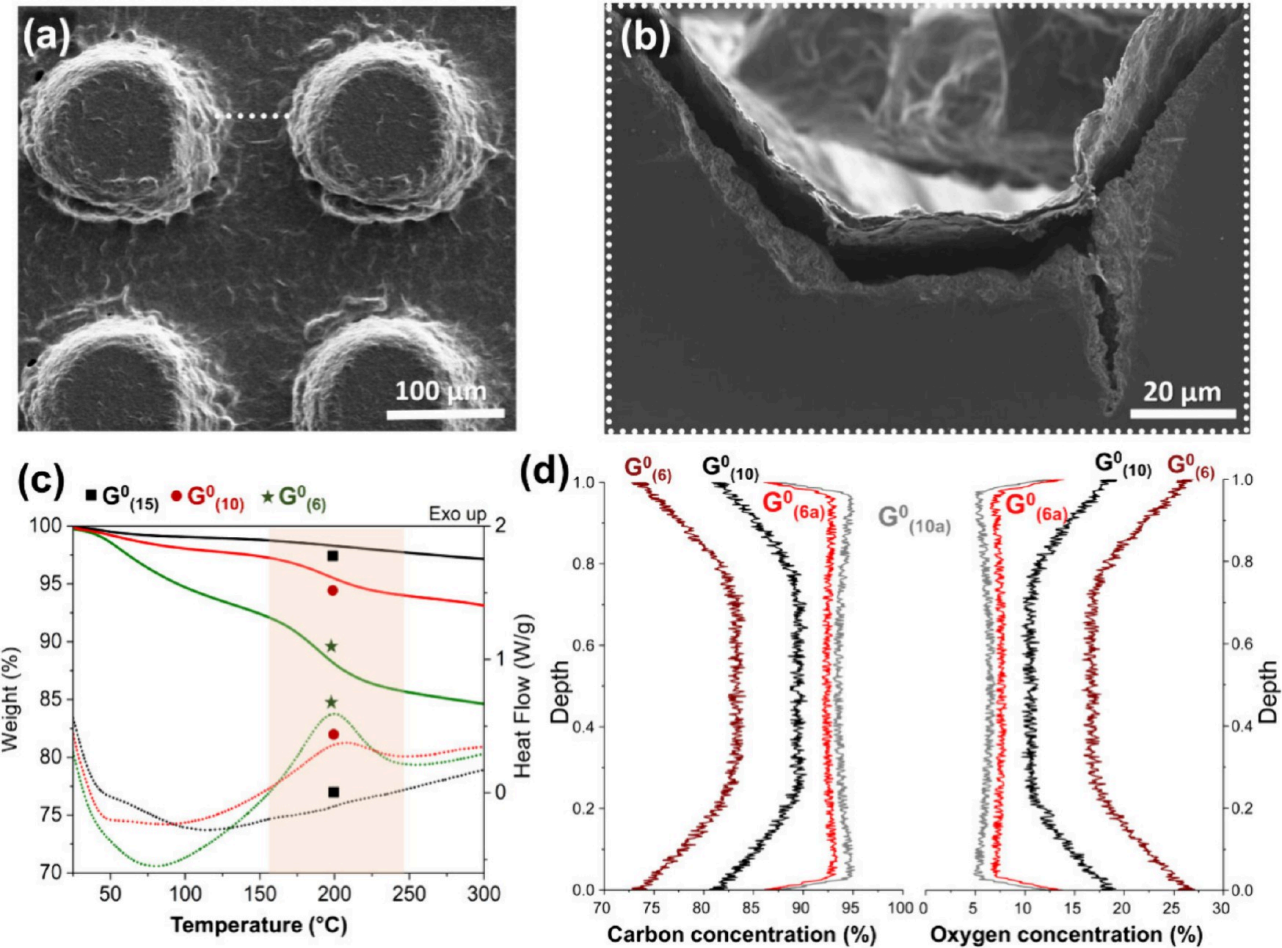
SEM images of the pillar-textured Si/SiO_2_ substrate with a cast G^0^_(6)_ film formed on its surface, before (a) and after (b) fracturing the substrate. The dotted white line in part a indicates the region between pillars where the substrate was broken and subsequently imaged. (c) Detailed TGA/DSC analysis of the G^0^ films with different degrees of oxidation, highlighting the exothermic transformation with an onset at ∼150 °C and the associated mass loss. (d) SIMS depth profiles demonstrating the C and O concentrations of G^0^ films with different levels of hydrolytic oxidation, before and after annealing, as a function of the film depth (in depth fraction).

The same coated array can subsequently be covered with an additional layer of SiO_2_, confining the film within a dielectric/conductive/dielectric layered architecture, as we demonstrate elsewhere.^24^ This architecture approximately mimics the coating of a thermal heat-sink region in a chip stack within a “system-on-Package” (SoP) electronics platform^53^ and the *in situ* preparation of a G^0^ thermally conductive film to dissipate the heat produced by tightly packed transistors in SoP.

#### 3.2.6 Mild Annealing and Film Cross-Linking

The specific structure of G_eh_, including its functional homogeneity and regioselectivity, produces an unusually strong degree of film organization, yielding G^0^ films with excellent transport properties. However, well-defined percolation and anisotropic metallic behavior are only observed after thermal treatment; thus, their thermal transformations were further investigated.

When annealed, G^0^ films undergo defined transformations, with onset temperatures around ∼150 °C, leading to significant changes in their structure and composition. The exothermic transformation, with a maximum at ∼200 °C (up to 111 J/g for G^0^_(6)_, as measured by DSC), which is accompanied by a corresponding mass loss at the same temperature (up to 3 wt % loss for G^0^_(6)_, as measured by TGA) (Figure 5c), is the result of condensation reactions among the functional groups.^54^ G^0^_(15)_ appears to be at the lower limit of hydrolytic oxidation required for cross-linking, as it presents an almost negligible thermal transition by DSC and mass loss by TGA. Most of the G^0^ functionalities are hydroxyl groups, forming predominantly ether bonds among sheets after solvent evaporation. Different from GO, G^0^ contains only residual carboxyl groups generated during heating,^55^ which results in a low abundance of ester and anhydride bridges (Figure 3a and Table 6). The mass loss observed by TGA can be directly associated with the evaporation of surface-adsorbed water (<150 °C), followed by interstitial water (>150 °C), and, less frequently, other molecules such as ethyl alcohol, produced by the different possible mechanisms of these reactions.^56^ Once interstitial water evaporation begins, these condensation reactions occur concomitantly. This allows further structural ordering and cross-linking of the films, reinforcing them at significantly milder temperatures than those required for graphitization, while avoiding temperature-related limitations to their applications. After cross-linking the G_eh_ species into G^0^, they are highly thermostable, even under oxidative atmospheres, with thermo-oxidative decomposition temperatures exceeding 600 °C (Figure 3b). This provides a broad processing temperature window for their application without material decomposition.

Examples of the possible condensation reactions occurring during cross-linking are summarized in eqs 8–11. The dominance of hydroxyl groups in the structure leads almost exclusively to the reaction described in eq 8; however, we also consider other possible residual functional groups and the most common condensation reaction mechanisms (eqs 8–10), including Claisen and Dieckmann condensation reactions (eq 11).

8 $$\mathrm{R-OH}+R^{\prime }\mathrm{-OH}\to {\mathrm{R-O-R}}^{\prime }+H_{2}O$$

9 $$\mathrm{R-COOH}+R^{\prime }\mathrm{-OH}\to \mathrm{R-COO-}R^{\prime }+H_{2}O$$

10 $$\mathrm{R-COOH}+R^{\prime }\mathrm{-COOH}\to R\mathrm{-OCOCO-}R^{\prime }+H_{2}O$$

11 $$\mathrm{R-COO-}R^{\prime }+\mathrm{R-}\mathrm{COO-}R^{\prime }\to R\mathrm{-COC-}\mathrm{R-COO-}R^{\prime }+R^{\prime }\mathrm{-OH}$$

Following the TGA/DSC results, we annealed the films at 150–200 °C, without the application of vacuum or an inert atmosphere, thereby enabling open casting or hot pressing of large-area films (see Section 3.3). The annealing/cross-linking dramatically alters the film structuration, further improving several mechanical and transport properties (see Section 3.4).

The bulk elemental distribution of the films before and after annealing was investigated by ultralow-energy SIMS, allowing precise profiling of carbon and oxygen distributions as a function of film depth.^57^ Notably, the films exhibit well-defined differences in the C/O ratio between their outer and inner regions (Figure 5d). Before annealing, films with different levels of hydrolytic oxidation present significant variations in O content, both in their inner and outer layers. G^0^_(6)_ presents an approximately 2-fold lower C/O ratio at the outermost layers (C/O ∼ 3 at the surface), which increases in a defined gradient toward the innermost layers (reaching C/O ∼ 6 at ∼25% film depth). Similarly, G^0^_(10)_ also shows an almost 2-fold higher C/O ratio in the inner region compared to the surface, but with overall higher values (C/O ∼ 9 vs C/O ∼ 5, respectively).

Upon annealing, G^0^_(6)_ and G^0^_(10)_ become significantly more similar, and the O distribution gradients largely disappear in both cases, leaving only residual O in the inner layers (C/O ∼ 16 and C/O ∼ 19, respectively) and a thin outer region (<5% of the film depth) with approximately 3-fold higher O content, corresponding to C/O values of ∼6 and ∼7, respectively. This indicates the formation of films with a well-defined heterolayered structure, consisting of a core of percolated graphitic domains and mildly oxidized graphitic outer layers. This structure closely resembles that of metals such as copper, which form a stable oxide layer at relatively low temperatures.^58^ Such an architecture, combined with high structural anisotropy, may contribute to the extreme conductivity anisotropy observed in these materials.^25^

### 3.3 Stability and Processability

After the functionalization reactions, free-standing films with well-defined structures are readily and reproducibly obtained. All characterization presented in this study was performed with films prepared in open air, dried at room temperature, and annealed without any steric or mechanical constraints. In addition to the aforementioned free-standing films, a vide variety of other structures can be easily obtained, including films cast onto complex texturized surfaces, as well as other products such as water-stable dispersions, highly concentrated and redispersible slurries, extruded or compressed films, pellets, and filaments (Figure 6a). The high stability in water and solvent mixtures enables film preparation even from highly concentrated dispersions (up to 4 wt %). Moreover, the strong anisotropy and sheet assembly, associated with a lower solvent content, allow for the rapid preparation of large-area films without disrupting the film structure.^24^ Importantly, annealing/cross-linking is performed without the use of vacuum or an inert atmosphere, as the thermal decomposition temperature (*T*_d_) of G_eh_/G^0^ is substantially higher than the annealing temperature (Figure 3b vs Figure 5c). In addition, the presence of small amounts of water facilitates effective cross-linking by inducing antiplasticization of the films.^54^ Consequently, large-area films can be prepared both at room temperature and via hot processing (casting or pressing), and the film size can be expanded without intrinsic limitation, being constrained only by the dimensions of the casting or pressing support.

**Figure 6:**
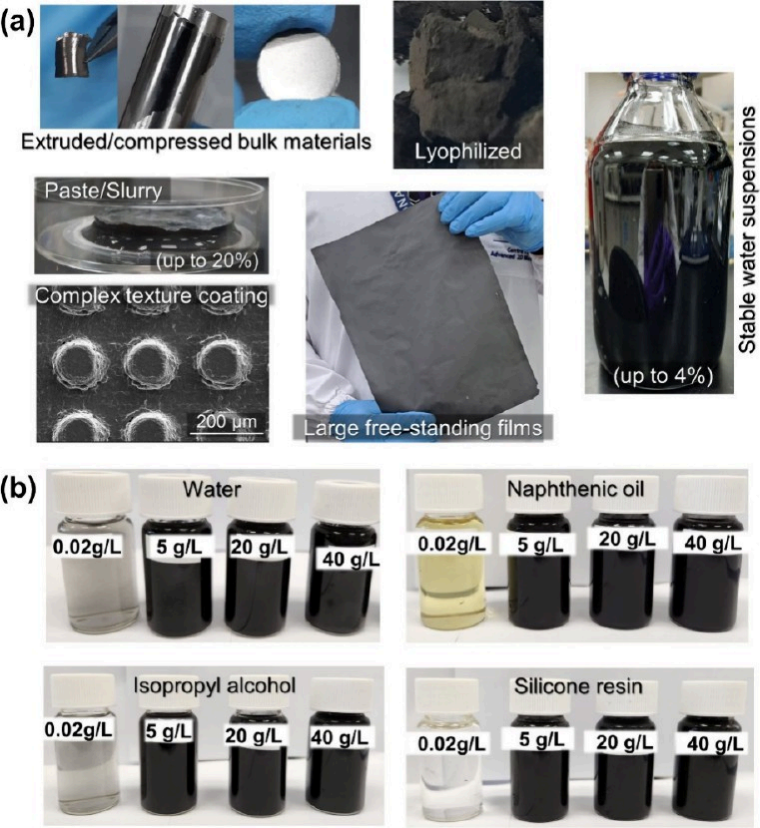
Processability and solvent stability. (a) SEM and photographic images of the products yielded by the G_eh_ platform, demonstrating the adaptability of the resulting materials to different target applications. Examples include G^0^ coatings on texturized/patterned complex surfaces, large (A4-sized) free-standing films, extruded bulk materials and pellets, highly concentrated aqueous suspensions, lyophilized powders, and highly concentrated pastes/slurries. (b) Photographic images of G_eh(6)_ dispersions, spanning more than 3 orders of magnitude in concentration, after 30 min of sonication, followed by 30 min of resting in hydrophilic and hydrophobic media of industrial interest.

Moreover, due to its amphiphilicity, in addition to its stability in water, G_eh_ also forms stable dispersions in most hydrophilic and hydrophobic solvents, oils, and resins commonly used in industrial formulations (Figure 6b). We observed no apparent concentration limit to this stability across the different media; in fact, stability is generally enhanced at higher concentrations, up to the gelation point. This behavior, combined with G_eh_’s amphiphilicity and edge-to-edge assembly, suggests soft glassy dynamics driven by self-stabilization, akin to those observed in highly amphiphilic clays such as laponite.^59^

### 3.4 Mechanical, Thermal, and Electronic Properties of Films

Due to the aforementioned processability, physicochemical properties, and ordered film assembly, G^0^ films exhibit a combination of mechanical, thermal, and electronic properties that clearly distinguish them from GO. Below, we summarize and discuss these properties, while the experimental setups are presented in the Detailed Characterization Methods section.

With regard to mechanical properties, micromechanical maps of the films before and after annealing, presented in Figure 7, are obtained using the same tip, RTESPA 300 (*k* = 40 N/m), to ensure better comparability. In addition, the films are also imaged using RTESPA 525 (*k* = 200 N/m) to obtain a more accurate determination of the average elastic modulus for the annealed sample, which presents a significantly higher elastic modulus (Figure 7).

**Figure 7:**
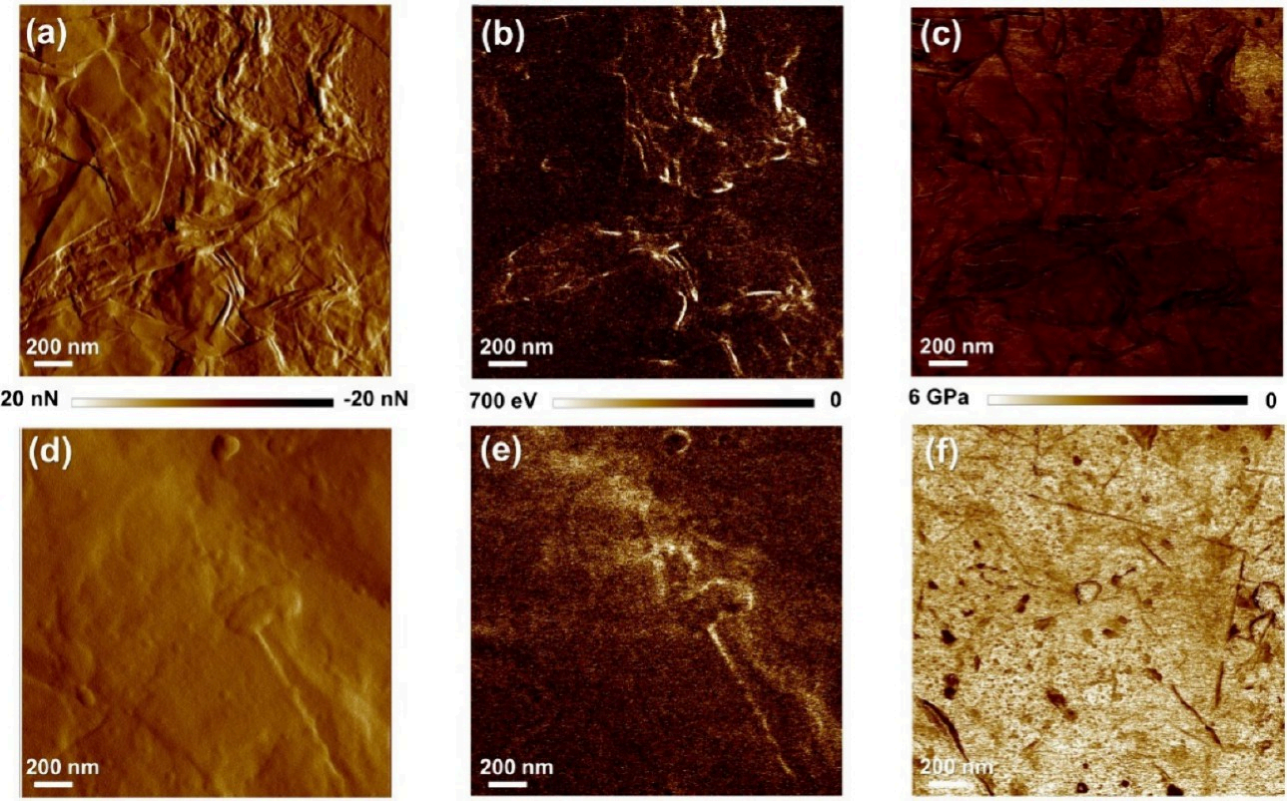
AFM micromechanical maps obtained by PeakForce-QNM, using an RTESPA 300 tip (*k* = 40 N/m), including (a and d) peak-force error, (b and e) dissipation, and (c and f) stiffness maps of G^0^_(6)_ films before (top row) and after (bottom row) annealing, respectively. The maps before and after annealing are normalized using the same scale for direct comparison.

Before annealing, the films display a structure of interacting sheets, in which fluctuations in mechanical properties near edge contacts allow for clear visualization of locally reduced stiffness (Figure 7a–c). These features are also visible in the peak-force error map, but only prior to annealing (part a vs part d in Figure 7). The same fluctuations cause mechanical energy loss and are clearly captured in the dissipation maps (part b vs part e in Figure 7). In these regions, the overall lower stiffness and distinct local minima in the micromechanical modulus (*E*′_m_) highlight the edge lines and corrugations (Figure 7c).

After annealing, a smoother film surface is observed, with sheet edge junctions or corrugations becoming virtually undetectable (Figure 7d,e). In addition, the disappearance of the moduli dips, a dramatic increase in stiffness beyond the measurement scale limit, and overall homogenization become evident after annealing (part c vs part 7 in Figure 7). We attribute this transformation to an extensive formation of covalent bonds via condensation reactions (see Section 3.2.6), leading to a type of “chemical stitching”^60^ that does not require any assembling medium, cross-linker, or catalyst.^24^

DMA of the films confirms the mechanical reinforcement after annealing. The storage (elastic) modulus increases approximately 2-fold upon annealing (from ∼10 to ∼21 GPa), while the loss (viscous) modulus changes only modestly (from ∼600 to ∼800 MPa). This indicates that the films are highly elastic, with a low degree of energy dissipation and low internal friction during deformation. The coefficient of thermal expansion (CTE) of the films also decreases by approximately factor of 2 after annealing, demonstrating strong structural fixation (see Figure 6 in ref 24). The combined mechanical behavior, including the micromechanical response (Figure 7), resembles that of densely cross-linked polymer networks,^61^ in which the structure becomes fully percolated after cross-linking. However, unlike polymer networks exhibiting a glass transition temperature (*T*_g_), these materials possess a negative CTE and are therefore annealed under tension (i.e., in a stretched conformation), leading to an order–disorder transition at low temperatures (<150 K). This transition causes a plethora of effects on their structure and properties, which we discuss elsewhere (see Figure 4f in ref 25).

Concerning the thermal and electronic transport properties of the films, these have been extensively discussed in our previous reports.^24,25^ Briefly, their in-plane thermal and electrical conductivities are comparable to those of conventional metals (*k* ≈ 180 W/mK and σ ≈ 300 kS/m), together with a low emissivity (ε ≈ 0.03). In contrast, the through-plane conductivities exhibit polymer-like values (*k*_r_ ≈ 0.2W/mK and σ_r_ ≈ 3.8 S/m), resulting in exceptionally high thermal and electrical anisotropies (ρ_*k*_ ≈ 10^3^ and ρ_σ_ ≈ 10^5^). These results are among the largest reported to date, with ρ_*k*_ comparable to that of randomly interlayer-stacked MoS_2_ ^62^ and ρ_σ_ comparable to ReS_2_ heterostructures.^63^ Unlike conventional metals, however, the density of G^0^ films remains only slightly above that of water (∼1200 kg/m^3^; for films cast in open air at room temperature, see Table S1), lying between those of GO and rGO^64^ and rendering them exceptionally light metallic materials. The experimental setups and measurement procedures used to obtain these values are described in detail in the Detailed Characterization Methods section.

## 4 Conclusions

Edge-hydrolyzed graphene was herein demonstrated to represent a distinct class of 2D graphitic materials, fundamentally different from both pristine graphene and GO. These materials are produced by harnessing the specific role of water in oxidative reactions,^15,16^ effectively transforming the process into an oxidative hydrolysis. Additionally, this reaction exploits the edge selectivity of ^•^OH species during the reaction with graphene, yielding a material with defective and oxidized edges while preserving an intact basal plane. The resulting 2D sheets are intrinsically amphiphilic and exhibit a pronounced tendency to self-assemble.

Through self-assembly, edge-hydrolyzed graphene forms highly ordered, polycrystalline films with tightly bound edge junctions. This architecture enables the retention of pristine graphene properties, while also facilitating their propagation across interconnected 2D domains, ultimately producing a percolated 3D network. The resulting films combine a smooth, metallic-like surface with high stiffness (*E* > 10^10^ Pa), flexibility, and bendability, while maintaining structural integrity across a wide thickness range. Moreover, preservation of the basal plane ensures graphene’s high thermal stability in both oxidative and inert environments, alongside a small (and negative) CTE, which prevents large film displacement and structural disorder upon heating. The ordered assembly and pronounced structural anisotropy of the films give rise to extreme contrasts in transport properties, rendering them metal-like conductors in-plane and polymer-like insulators through-plane. Taken together, these findings establish edge-hydrolyzed graphene assemblies as a novel material platform, whose distinctive properties, summarized in Table S1, highlight a field that remains at an early stage of exploration.

## Supplementary Material





## Data Availability

The data that support the findings of this study are available from the corresponding authors upon reasonable request and will be deposited on ChemRxiv (10.26434/chemrxiv-2024-h3pkk-v3).

## Notes

SIMS oxygen calibration curve (Figure S1), averaged TGA and DSC curves with standard deviation from triplicate measurements (Figure S2), G0 film properties summary (Table S1), thermal transport setups (Figure S3), and electronic transport setups (Figure S4) (PDF)
Fast casting of G_eh(6)_ at 60 °C (Video S1) (MP4)
