# Supplementary material for: Engineering Disordered Metallic Carbonaceous Materials: A Protocol for the Synthesis via Graphene Edge Hydrolysis
Source: ACS Appl Nano Mater. 2026 Feb 27;9(10):4699–714. doi: 10.1021/acsanm.6c00047 (PMC12993809; doi:10.1021/acsanm.6c00047)
Supplement: Supplementary file 1 [file an6c00047_si_001.pdf]

Supporting Information (SI)

Engineering Disordered Metallic Carbonaceous Materials: A Protocol for the Synthesis via Graphene Edge-Hydrolysis

Katarzyna Donato[1,2], Gavin Kok Wai Koon[1,3], Sarah Lee[1], Alexandra Carvalho[1,3], Hui Li Tan[1], Mariana Costa[1,3,4], Paweł Piotr Michałowski[5], Zuzana Němečková[6], Petra Ecorchard[6], Ricardo K. Donato[6], Antonio Castro Neto[1,3,4,7*]*

[1] Centre for Advanced 2D Materials, National University of Singapore, 117546, Singapore.
[2] J. Heyrovský Institute of Physical Chemistry, Czech Academy of Sciences, Dolejškova 2155/3, Prague 182 23, Czech Republic.
[3] Institute for Functional Intelligent Materials (I-FIM), National University of Singapore, 117544, Singapore.
[4] Department of Materials Science and Engineering, National University of Singapore, 117575, Singapore.
[5] Łukasiewicz Research Network, Institute of Microelectronics and Photonics, 02-668 Warsaw, Poland
[6] Institute of Inorganic Chemistry of the Czech Academy of Sciences, Husinec-Řež 1001, 250 68 Řež, Czech Republic.
[7] Department of Physics, National University of Singapore, 117551, Singapore.

E-mail: donato@iic.cas.cz; c2dhead@nus.edu.sg

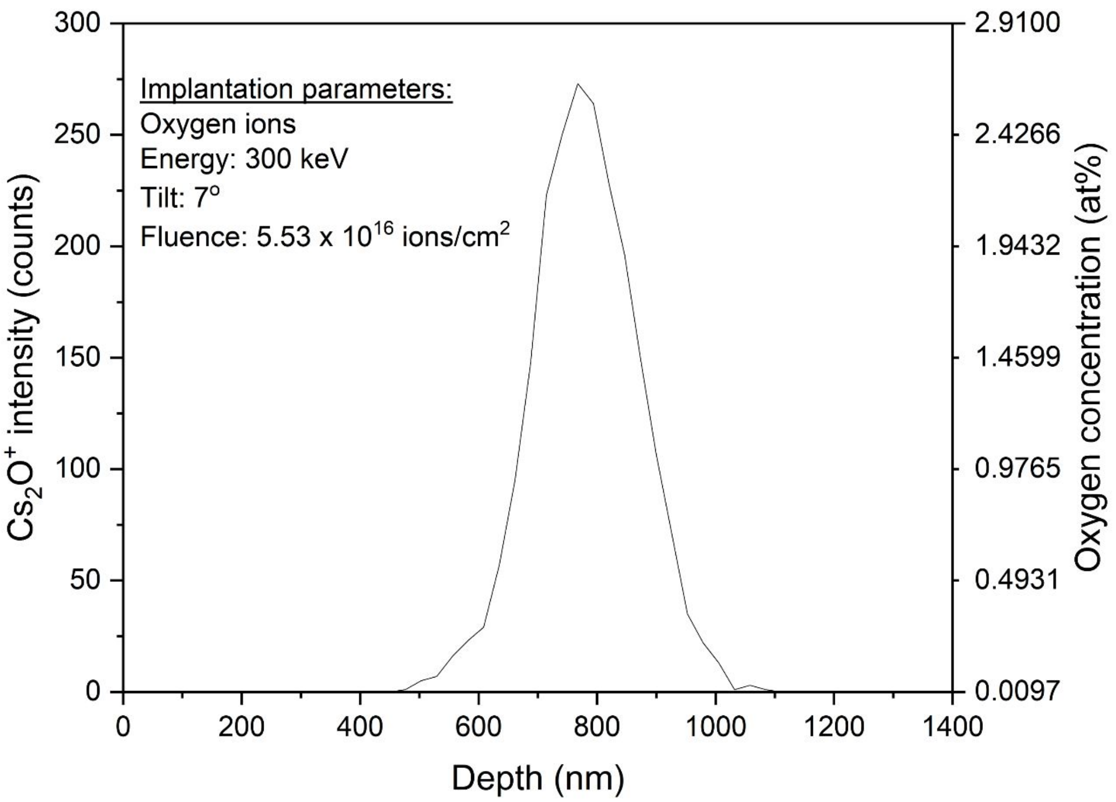

Figure S1. A representative oxygen implantation profile on a HOPG reference, for oxygen calibration.

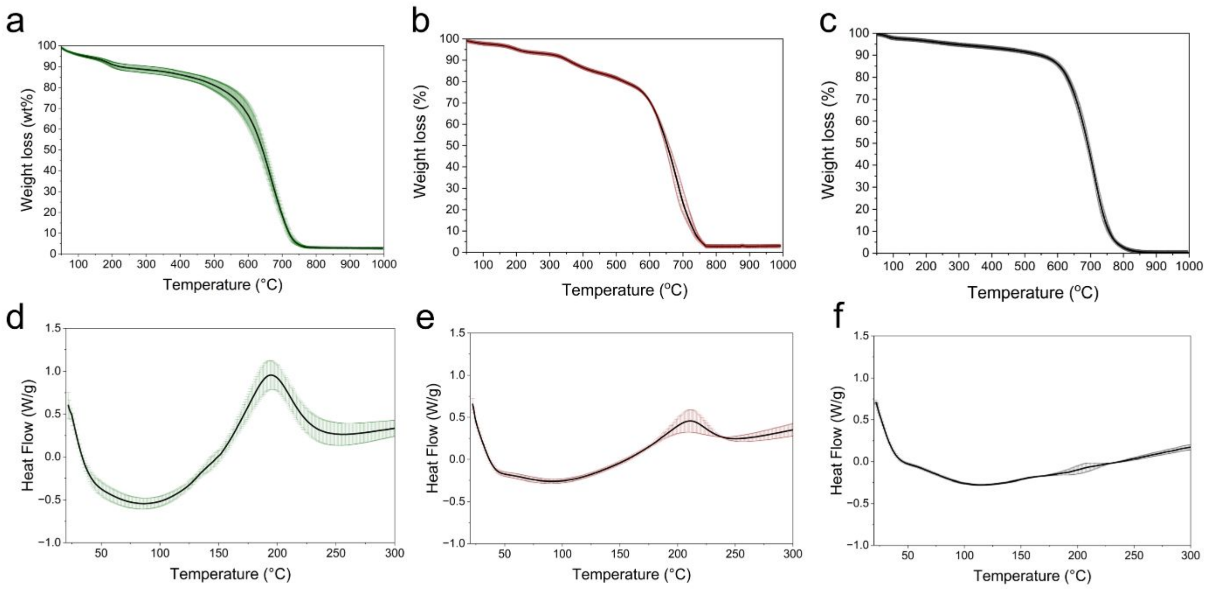

Figure S2. TGA and DSC averaged curves with standard deviations from triplicate measurement of $G_{eh(6)}$ (a and d), $G_{eh(10)}$ (b and e), and $G_{eh(15)}$ (c and f). The averaged TGA curves were used in Figures 3 and 5, and the averaged DSC curve was used in Figure 5.

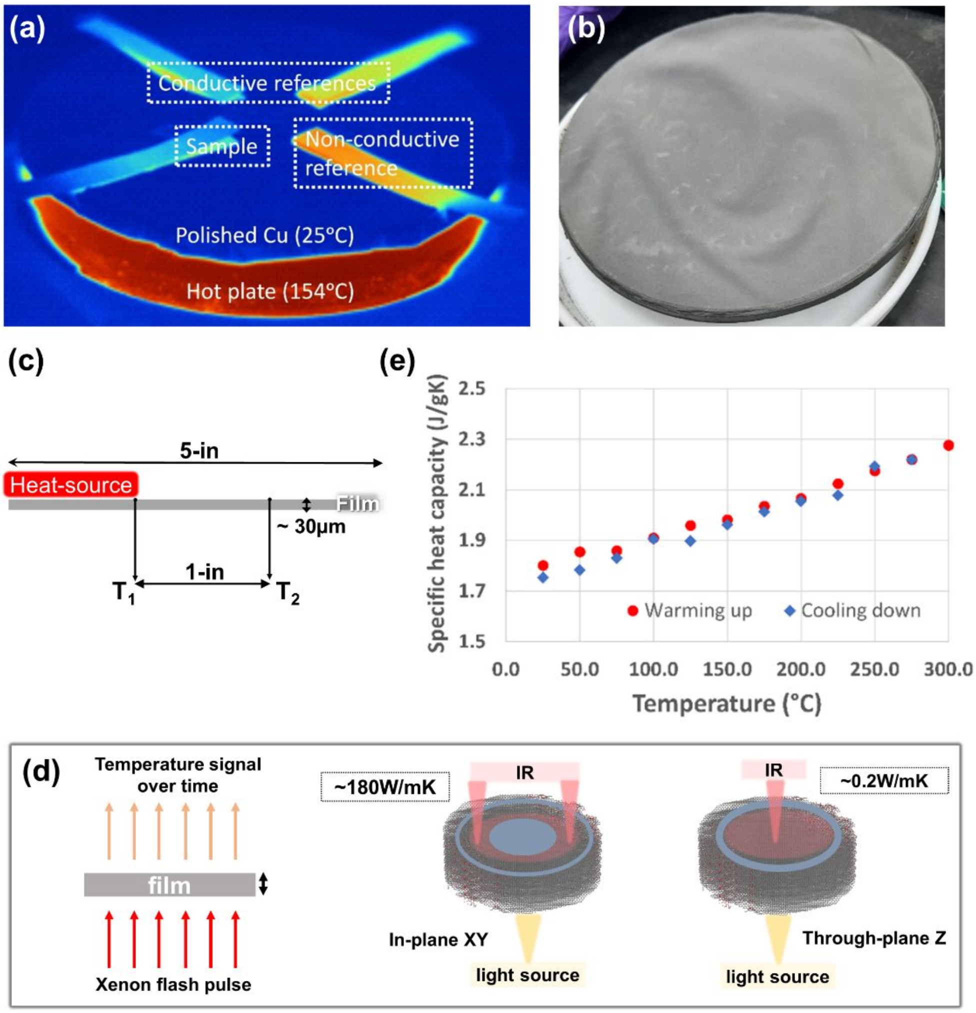

Figure S3. Thermal transport characterizations. (**a**) Thermal image showing the setup used observe thermal radiation of conductive and non-conductive samples, also used to obtain the images in Figure 6 of Reference[1]. (**b**) Photographic image of the film with 5 inches in diameter and ~50 μm thickness used to obtain the thermal image of Figure 6 in Reference[1]. (**c**) Schematic drawing showing the setup to measure the conductive heat values used to calculate ε, where a heat source T_1 is heated to 200 °C and temperature readings are made in T_2. (**d**) Schematic drawing showing the light flash technique used for measuring the thermal diffusivity of the films. Free-standing samples with thickness varying between 50-400 μm were pre-cut to a circular shape with a diameter of 1 inch, to be fitted into the pre-defined sample holders. (**e**) Specific heat capacity (C_p) measurement of $G^0_{(6a)}$, used for determining the thermal conductivity of the films.

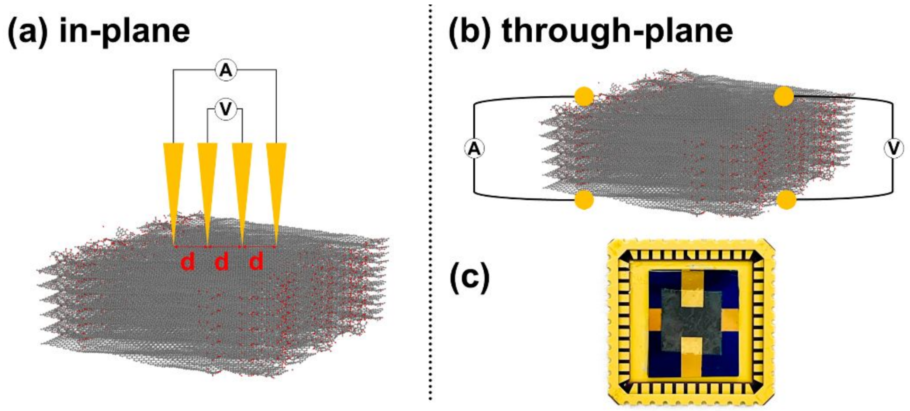

Figure S4. Schematic representations showing the four-point probe measurement setups. (**a**) In-plane measurement with four electrical contacts with equal spacing *d* in a line, where the current *I* is applied on the outer two probes and the resultant voltage ΔV drop is measured between the inner two probes. (**b**) Through-plane measurement with two electrical contacts on the top and two on the bottom of the film. (**c**) A photographic image of the actual device represented in (**b**).

Table S1. Reproducible (based on at least three repeated measurements) and scalable (for gram-scale samples) properties of $G^0_{(6a)}$ films.

Physical	Flexible, bendable film
Color	Metallic, shiny
Typical thickness range (μm)	1-400
Density (kg/m^3)	~1,200
[1]Electrical conductivity (S/m) (In-Plane/Through-Plane)	320,000/3.8
[2]Thermal conductivity (W/mK) (In-Plane/Through-Plane)	180 / 0.16
[3]Max. anisotropy measured (Electrical/Thermal)	84,210 / 1125
[4]Specific heat capacity (Jkg^{-1}K^{-1})	1,700-2,000
[5]Coefficient of thermal expansion (CTE, ppmK^{-1})	-4×10^{-6}
[6]Young's modulus (Pa)	2×10^{10}
[7]Stability (K)	> 780 (oxidative) and > 1300 (inert)

[1]The conductivity values were measured at room temperature (298 K) in a cryostat system using both the in-plane and through-plane device configurations;
[2]The conductivity values were measured at room temperature (298 K) via the laser flash analysis method;
[3]The anisotropy values were calculated based on the ratio of the in-plane conductivity to the through-plane conductivity;
[4]The specific heat capacity value was obtained with the comparison method with a graphite reference sample via laser flash analysis;
[5]The coefficient of thermal expansion was measured on annealed sample using a TMA system;
[6]The Young's modulus was measured with the tapping mode of an atomic force microscope (AFM) system;
[7]Stability tests were conducted by annealing the sample at temperatures up to 1100 °C.

Reference

(1) Donato, K. Z.; Koon, G. K. W.; Lee, S. J.; Carvalho, A.; Tan, H. L.; Costa, M. C. F.; Tolasz, J.; Ecorchard, P.; Michałowski, P. P.; Donato, R. K.; Castro Neto, A. H. Disordered Metallic Carbon Materials from Graphene Edge Chemistry. *Mater. Today* **2024**, *79*, 49–59. https://doi.org/10.1016/j.mattod.2024.07.011.
